# Pregnancy rates in hair sheep after Ovsynch synchronization and a combined intracervical fixed-time artificial insemination and 10-day mating period

**DOI:** 10.14202/vetworld.2019.1779-1783

**Published:** 2019-11-15

**Authors:** D. A. Vallejo, J. D. Londoño, Y. A. Yepes, V. Tamayo, A. F. Mejia, J. G. Maldonado

**Affiliations:** 1 Department of Theriogenology, School of Veterinary Medicine, OHVRI Research Group, Faculty of Agrarian Sciences, University of Antioquia, Medellin, Colombia; 2 National Learning Service – SENA, Center of Renewable Natural Resources – La Salada, Research Group La Salada, Colombia Government, Caldas, Colombia

**Keywords:** ewes, fixed-time artificial insemination, Ovsynch, pre-synch

## Abstract

**Aim:**

This study aimed to evaluate the pregnancy rates in hair ewes using an Ovsynch synchronization protocol under a breeding system that combines fixed-time insemination plus a 10-day mating period as an alternative.

**Materials and Methods:**

Through an experimental study (n=27), ewes were randomly located into one of three treatments: (1) Pre-synch (n=9): Prostaglandin F2α (PGF2α)+Gonadotropin-releasing hormone (GnRH)+PGF2α+GnRH; (2) Ovsynch (n=9): GnRH+PGF2α+GnRH; and (3) control: Ewes bred by natural mating (NM) (n=9). Ewes were fixed-time inseminated (fixed-time artificial insemination [FTAI]) with fresh semen, collected just before the insemination time through vaginoscopy at 16 h after the second GnRH (gonadorelin) injection. Each experimental group was placed separately during 15 days and, after this time, fertile rams were allowed back with ewes for a 10-day mating period. Control group ewes remained with the rest of the herd suitable for breeding and were bred under NM. Pregnancy diagnosis was performed by ultrasound at 28-, 56-, and 84-day post-breeding to differentiate between FTAI and NM pregnancies. Total (FTAI±NM) pregnancy rates at 56-day post-breeding were used to compared Pre-synch, Ovsynch, and control. For this purpose, two-tailed proportions comparison z-test was used with a 95% confidence level, for testing as the null hypothesis whether two proportions were equal.

**Results:**

Pregnancy rates were higher in control ewes (66.4%) than FTAI (46.6%). When pregnancy rates after a 10-day mating period (40%) were added, the final rate (86.6%) was significantly (p<0.05) higher in Ovsynch-based protocols. The pregnancy rate was significantly lower in FTAI ewes compared to FTAI +10-day mating group (p<0.05). The overall pregnancy rate was 88.0, 85.7, and 67.0 (p>0.05) for Pre-synch, Ovsynch, and control ewes, respectively.

**Conclusion:**

These results provide evidence on the benefits of combined FTAI protocols for improving the reproductive efficiency of sheep.

## Introduction

There are several reasons to support why producers ask for the establishment of protocols for controlling estrus and ovulation in ewes. The opportunity to use artificial insemination (AI) is advantageous in the ovine industry, as it significantly speeds up the progression of genetic merit and synchronizing the lactation period of groups of dams that can later be managed as a batch, in order to overcome the need to detect estrus or schedule natural mating (NM) when having problems with the ram or the reproductive performance of females [1,2]. The progesterone impregnated intravaginal sponges, left *in situ* for 12-17 days in the breeding season or the use of the progesterone-impregnated vaginal device (polyurethane sponges or controlled internal drug release devices), with an injection of equine chorionic gonadotropin at device removal, are widely used methods. Although both options can give high or acceptable rates of synchronization and lambing, they have some disadvantages of being applied for an extended period. One of them is the high costs for the treatment of a large number of animals. Other is the incidence of vaginal mucosa inflammation with discomfort and purulent discharge induced by the progesterone sponge [3,4]. In addition, it was reported that a prolonged time of administration could result in low conception rates, probably as a result of impaired sperm transport *in vivo* or an ECG immunogenic reaction associated with low fertility rates in ewes [2,5,6]. Gonadotropin-releasing hormone (GnRH)-based protocols were proposed as an alternative for providing a source of P4 for inducing ovulation or luteinization of follicles. In cows, a hormonal regimen that includes the administration of a GnRH-PGF2-GnRH treatment known as “Ovsynch” protocol was proposed to control the follicular waves as well as the corpus luteum lifespan [7]. While different studies demonstrated the success of GnRH-based protocols in synchronizing and inducing estrus in cows and goats, fewer studies have examined its use in ewes [2-5,8] and the effect of differences in time, dosage, and GnRH schedule. Furthermore, there is scarce information related to the efficiency of these protocols when used in out-of-season breeding in ewes from tropical herds.

The estimated population of sheep in Colombia is near to 3.4 million animals (74% of the total population of small ruminants) with a population increase of 2.04% in the past 5 years, becoming a vital element for the growth of the livestock sector in the country. Ovine livestock systems from high-altitude tropics in Colombia are based on open grazing systems with an energy supplementation twice per day, in altitudes ranging from 1500 to 3000 m; average rainfall of 500-2000 mm/year, and a temperature range from 3 to 18°C [9-11]. Regarding reproductive management, continuous free mating programs are used in 82% of the herds, <3% of the herds use AI, and the average females ram ratio is 30:1. Herds have open days ranging from 91 to 150 days, and reproductive parameter-based decisions, hormonal treatments, or ultrasound pregnancy diagnosis are not current practices [11]. While such characteristics of sheep-producing systems predominate in high-altitude tropical systems and exhibit acceptable fertility due to low dependence of seasonal effects, breeders are interested in alternative strategies that allow them to improve the reproductive efficiency of their herds.

This study aimed to evaluate the pregnancy rates in ewes subjected to GnRH-based synchronization protocols under a breeding system that combines fixed-time AI (FTAI) plus a 10-day mating period as an alternative to NM.

## Materials and Methods

### Ethical approval

This study was conducted by the experimental practices and standards approved by the Institutional Animal Care and Use Committee, SENA – Research Center of Renewable Natural Resources, Colombia Government. All efforts were made to minimize animal suffering.

### Animals and experimental design

An experimental study was conducted at the Research Center of Renewable Natural Resources – “La Salada,” Antioquia, Colombia. The center is a part of the National Learning Service – SENA, of the Colombian Government. The herd is located at an altitude of 1900 m, an average temperature of 18°C, 85% humidity, in a very humid premontane forest (Holdridge classification system). For the experiment, 27 ewes (Dorper and Katahdin) with an age range of 3-4 years were used. Selected animals were clinically healthy at the semiological examination and had given birth at least once before the beginning of the study. Animals were grouped by breed and, through a completely randomized design, randomly located (nine animals per group) into one of three treatments: (1) Pre-synch: Prostaglandin F2ɒ – GnRH – Prostaglandin F2ɒ – GnRH; (2) Ovsynch: Gonadotropin-releasing hormone – Prostaglandin F2ɒ – gonadotropin-releasing hormone; and (3) NM (control group).

### Housing and feeding system

Animals were maintained in separated lots, in an open grazing system, grazing *ad libitum* on *Cenchrus clandestinum* (Hochst. ex Chiov.) Morrone grass. This pasture was managed with a harvest period ranging from 25 to 45 days according to the raining periods. In addition, according to the NRC recommendation for sheep [12], animals were fed with 0.5 kg of commercial supplementation twice per day, and *ad libitum* access to water and minerals supplemented salt.

### Inclusion criteria

All ewes that had given birth at least once were evaluated by ultrasonography through B-Mode and color Doppler ultrasound (Mindray^®^ Z5 Vet, Shenzhen, China). Inclusion criteria were as follows: (a) Clinically healthy at reproductive examination, (b) non-pregnant, (c) exhibiting normal cyclicity as assessed by the presence of corpus luteum and follicular dynamics during three consecutive reproductive examinations, 7 days apart, and (d) clinically healthy all along during the experiment.

### Hormonal treatments

**Figure-1:**
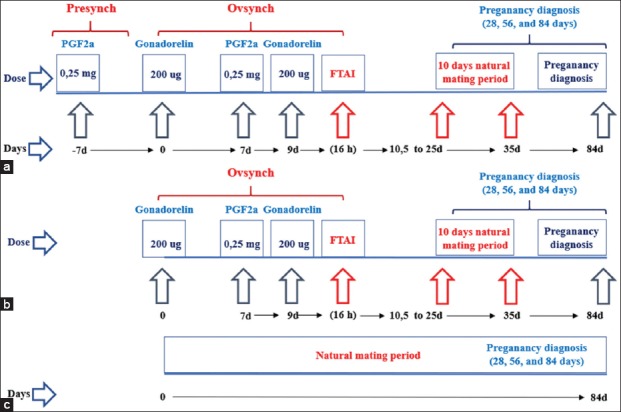
Experimental schedule of the study. a: Pre-synch+Ovsynch protocol. b: Ovsynch protocol. c: Control group.

Selected animals were randomly allotted to one of two treatments (Figure-1): (1) Animals of “Pre-synch Group” that received an i.m. injection of 0.25 mg of the PGF2a analog cloprostenol (1 ml^®^, MSD Animal Health, USA) 7 days before the beginning of the protocol, then, on day 0, an i.m. injection of 200 ug of gonadorelin acetate (2 ml Fertagyl^®^, MSD Animal Health, USA), followed, 7 days later, by 0.25 mg of cloprostenol i.m and, on day 9, the second injection of 200 ug of gonadorelin, and FTAI 16 h later. (2) Animals of “Ovsynch Group” received on day 0, 200 ug i.m. of gonadorelin, followed, 7 days later, by 0.25 mg of cloprostenol i.m and, on day 9, the second injection of 200 ug of gonadorelin, and FTAI 16 h later. Control animals were left to mate naturally with no hormonal treatment. All treatments were scheduled to finish on the same day, and no further treatments were applied following the synchronization protocols.

### Semen collection and straw preparation

Animals were inseminated only once at a predetermined time, done 16 h after the second GnRH (gonadorelin) injection. Semen was collected just before insemination time using an electroejaculation device (ElectroJac^®^ 6+Ram/Boar probe). After collection, semen motility, concentration, and morphology were evaluated. Ejaculates with >80% initial progressive motility, sperm concentration >750 million/ml, and >70% of normal morphology were selected and diluted (Andromed^®^, Minitube, Germany) to a concentration of 500×10^6^ sperm/ml and packaged into 0.25 ml straws (Mini-Straw, clear, 0.25 ml, Minitube^®^, Germany). Two rams of known fertility were used as semen donors (one animal per breed).

### FTAI

FTAI was carried out through vaginoscopy by a Disposable Vaginal Speculum (Duckbill 200 mm, Medical Plus^®^, Argentina) using an AI gun (Quick-Lock AI Instrument, Minitube^®^, Germany) to deposit 0.25 ml of diluted semen inside the cervix. Immediately after the AI, each one of two FTAI group was placed separately for 15 days. After this time, males were allowed in contact with females for a 10-day mating period. During the experiment, the control group remained with the rest of the herd and was formed by the females suitable for breeding, with no genetic selection, and under general reproductive management (open mating system).

### Assessment of pregnancy outcome

Transvaginal and transrectal B-mode ultrasound was used to pregnancy diagnosis [13,14] using Mindray^®^ Z5 Vet (Shenzhen, China) equipped with sectorial and transrectal 5-7.5 MHz probes. For pregnancy diagnosis, additional color Doppler ultrasound was used for the assessment of corpus luteum function [15]. The Doppler settings used were 20% color gain, 1.0 kHz pulse repetition frequency, 7 cm of depth, and 75 MHz wall filter. Ultrasound examinations were performed at 28-, 56-, and 84-day post-breeding to differentiate between AI and NM pregnancies. Total (AI+NM) pregnancy rates at 56-day post-breeding were used to compared Pre-synch, Ovsynch, and control.

### Statistical analysis

Data were analyzed by descriptive statistics. Two-tailed proportions comparison z-test was used for testing with a 95% confidence level, whether two proportions were equal as the null hypothesis.

## Results

**Figure-2:**
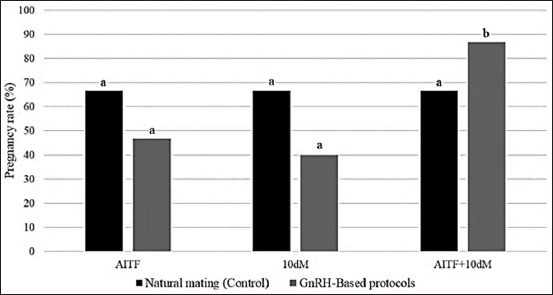
Pregnancy rates in ewes after gonadotropin-releasing hormone (GnRH)-based protocols, cervical fixed-time artificial insemination, and 10-day mating period (Natural mating vs. GnRH-based synchronization). Natural mating=Control group pregnancy rate, GnRH-based protocols=Global pregnancy rates merging Pre-synch and Ovsynch protocols, AITF=Fixed-time artificial insemination, 10-day M=10-day mating period, NP=No pregnant, Total=Total pregnant rates. Different letter indicates a t-test significant difference (p<0.05).

**Figure-3:**
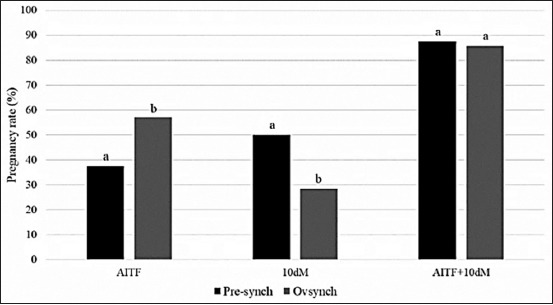
Pregnancy rates in ewes after gonadotropin-releasing hormone (GnRH)-based protocols, cervical fixed-time artificial insemination, and 10-day mating period. (Pre-synch vs. Ovsynch). AITF=Cervical time-fixed artificial insemination; 10-day M=10-day mating period; NP=No pregnant; Total=Total pregnant rates. Different letter indicates a t-test significant difference (p<0.05).

During the study, all treatments, procedures, and follow-up were developed successfully without unforeseen. Three ewes were excluded due to systemic disease (two ewes) or reproductive disease (one ewe). In total, 15 ewes were synchronized, 46.6% (n=7) get pregnant after AITF and 40% (n=6) get pregnant after the 10-day mating period. The pregnancy rate in the control group was 66%. After the combination of intracervical FTAI and 10-day mating period, the pregnancy rate was 86.6%. The pregnancy rate of the GnRH-based protocols (global pregnancy rates merging Pre-synch and Ovsynch protocols) was 20.6% higher (p<0.005) compared with the control group (Figure-2). All animals continue pregnant at the 84 post-breeding days. In this study, a pregnant rate >80% was found in both Pre-synch and Ovsynch groups. However, the proportion of ewes pregnant during the AITF (Figure-3) was 20% higher in Ovsynch group than the pre-synchronized group.

## Discussion

The results of the study show expected results, in which pregnancy rates in natural breeding (control group 66.4%) were higher than AITF. However, when pregnancy rates after a 10-day mating period were added, final results were significantly (p<0.05) higher compared to the NM, considerably increasing the efficiency of the GnRH-based protocols (Pre-synch – 88%; Ovsynch – 85.7%).

In this study, a pregnant rate >80% was found in both Pre-synch and Ovsynch groups. However, the proportion of ewes pregnant during the AITF was 20% higher in Ovsynch group than the pre-synchronized group. The results suggest that the use of PGF2ɒ as a pre-synchronization method before Ovsynch protocol in cycling ewes does not increase the pregnancy rates after AITF. This result could be explained by two ways considering that Ovsynch protocol at specific stages of the estrous cycle has been associated with reduced pregnancy success: (1) A low response of PGF2 injection, with corpus luteum remnants at the initiating of treatment resulting in less fertile oocytes being ovulated [16] and (2) luteolysis after PGF2 injection followed by ovulation and a lower concentration of progesterone at the initiating of treatment with variable response to the first GnRH injection resulting in not synchronized estrus [17]. Considering that sample size is the main limitation of the study, we recommend further research using a higher sample size to assessed pregnant rates in ewes treated with Ovsynch or Pre-synch protocols.

Few studies using GnRH-based synchronization protocols have been performed in ewes. The results of the present study showed a PR of 57% in cycling ewes treated with the Ovsynch protocol and intracervical AITF. Similar to the results of the present study, a study performed in Rahmani ewes in Egypt, designed to compare different Ovsynch protocols and artificially insemination, found that using GnRH, 0 day; prostaglandin F2α (PGF2α) 7 days later; and GnRH 48 h later during the breeding season resulted in 60% lambing rate [2]. A study conducted in the highlands of Ethiopia in 90 adult cycling hair sheep found PR of 87.7% with “GnRH” treatment (days 0-6-9 sequence) and bred by NM [18]. Another study performed in Turkey found that overall onset/duration of estrus and pregnancy/lambing rates was markedly lower with the standard Ovsynch synchronization program than the long- and short-term progesterone sponge – ECG protocols in fat-tailed ewes synchronized during the breeding season [3]. In general, the results of the use of GnRH-based protocols are positives but different from what was reported by these authors, the present study was conducted outside of breeding season.

Several studies using PGF2a-based protocols have been reported [19-21], but no reports were found on the use of GnRH-based protocols. During the non-breeding season in Italy, a study designed to assess the effectiveness of short-term Ovsynch protocols (5-7 days) in lactating ewes exposed to fertile rams found similar to those of our study [5]. In Mexico, the use of the Ovsynch protocols and FTAI resulted in PR of 33 and 46% using 50 ug or 100 ug of GnRH, respectively [8].

The main limiting factor technique for using FTAI in ewes is the semen deposition through the cervix into the uterus. The results of the present study showed that intracervical FTAI is an efficient insemination method, practical, economical, and easy access for the breeders. The reported pregnant rates with cervical insemination range between 36 and 60% and depend on the deeply of semen deposition, the protocol, and time of insemination [22]. On the other hand, as described by Consentino [15], the use of color Doppler ultrasound to assess corpus luteum function increases the reliability of early pregnancy diagnosis, 28-day post-breeding, as it was performed in our study.

Finally, in our study, it was found an increased PR when adding a 10-day mating period to the FTAI. There is scarce information about similar studies, except the report of a lambing rate of 90% during the non-breeding (90%) seasons in Karakul ewes that combine ram effect with two injections of PGF2α 10-day apart plus an additional injection of GnRH before the first injection of PGF2α [23]. Under the management conditions of the sheep herds in Colombia, a breeding system that combines FTAI followed by a mating period appears as a promising method to increase the reproductive performance of ewes. Further research using a higher sample size is recommended.

## Conclusion

The “Ovsynch” protocol is an economical alternative that offers reasonable pregnancy rates in ewes from tropical herds. The breeding system that combines intracervical FTAI plus a 10-day mating period had a final pregnancy rate >80%, which can be used to improve the reproductive management and fertility of the herds. GnRH-based protocols are promising alternatives for both controlled NM and FTAI in ewes.

## Authors’ Contributions

DAV and JGM were involved in the study design, data analysis, and writing and editing of the manuscript. JDL, YAY, VT, and AFM were involved in the data collection and writing of the manuscript. All authors read and approved the final manuscript.
